# A Novel Design Method of an Evolutionary Mold Cooling Channel Using Biomimetic Engineering

**DOI:** 10.3390/polym15040798

**Published:** 2023-02-04

**Authors:** Jae Hyuk Choi, Jinsu Gim, Byungohk Rhee

**Affiliations:** 1 Department of Mechanical Convergence Engineering, Gwangju University, Gwangju 61743, Republic of Korea; 2 Department of Mechanical Engineering, University of Wisconsin–Madison, Madison, WI 53706, USA; 3 Wisconsin Institute for Discovery, University of Wisconsin–Madison, Madison, WI 53715, USA; 4 Department of Mechanical Engineering, Ajou University, Suwon 16499, Republic of Korea

**Keywords:** injection molding, biomimetic engineering, conformal cooling channel, Murray’s law

## Abstract

In this study, an evolutionary cooling channel, a new methodology for designing a conformal cooling channel, was proposed. This methodology was devised by imitating the way that a plant’s roots grow towards a nutrient-rich location. Additionally, Murray’s law was applied to increase the cooling efficiency through minimizing the pressure loss of the cooling water inside the cooling channel. The proposed method was applied to the specimen shape to verify the concept, and it was confirmed that efficient cooling was achieved by applying it to the headlamp lens cover part of an actual vehicle. When this methodology was applied, the temperature deviation of the part could be improved by about 46% in just third generations, and the pressure loss could be reduced by about 10 times or more compared to the result of applying the straight-line cooling channel.

## 1. Introduction

The injection molding process comprises the filling and packing stages of injecting a molten polymer into a mold and the cooling stage of the polymer material to eject the product. A higher amount of product can be produced under a higher cooling rate. Therefore, several previous studies have aimed to improve cooling efficiency [1,2,3]. However, since cooling channels are manufactured using conventional machining, only a straight line can be used as a cooling channel. It is difficult to achieve sufficient cooling efficiency with only straight-line-based cooling channels for recent curved products, especially for vehicle components or household appliances.

A Conformal Cooling Channel (CCC), which is created along the surface of the product has been proposed as a solution to increase cooling efficiency for curved products, as well as the resulting quality of injection-molded parts [4]. Since CCC is not limited by its shape, it is difficult to manufacture using the traditional machining method. However, recently developing metal 3D printing technologies has enabled the fabrication of CCC.

An initial CCC was proposed by Sach and Xu [5,6]. They prepared a design method for a CCC using the basic heat transfer equation. In addition, based on the proposed formula, the mold was manufactured and tested to show that the cooling efficiency was superior to that of conventional cooling channels. Unlike conventional cooling channels, the shape-adaptable cooling channel may form a cooling channel in a completely free form. Thus, further research is required to identify the appropriate structure.

Au constructed a CCC using the scaffolding architecture that connects several squares with sides of several mms [7]. Wang divided the product’s surface into certain areas using a Centroidal Voronoi Diagram (CVD) and fabricated a cooling channel along the interface [8]. Both approaches were pioneering methods for installation, but the practicality was low because the flow stagnation was not considered. Gao suggested a machine-learning-aided CCC design method [9]. Oh and Kuo proposed triply periodic minimal surface (TPMS) structures and hybrid fillers to improve cooling efficiency using CCC, but they did not also consider the flowability in the cooling channels [10,11].

To solve this problem, we aim to develop a method to minimize flow stagnation and propose the shape of a new cooling channel with high cooling efficiency. In addition, we aim to increase applicability by developing a shape-adaptable cooling channel that can overcome practical limitations. Flow stagnation induces not only lower cooling efficiency resulting in severe warpage but also higher corrosion problems inside of the cooling channels resulting in maintenance and repair issues. Therefore, we imitated the structure and growth method of plant roots from the nature for the design of CCC.

Flow stagnation does not occur inside plant roots, which evolve into nutrient-rich zones. Ding and Wechsatol confirmed that cooling performance could be maximized while minimizing pressure drop using a tree-shaped network that mimics the branching rule of plant branch growth to improve heat transfer efficiency during cooling [12,13].

In this study, a cooling channel mimicking plant roots was installed in a hot region, and the amount of heat emitted from the product was approximated as the nutrients in the soil. To imitate the root shape of a plant, the cooling channel was fabricated in a bifurcation pattern according to Murray’s law [14,15]. The amount of heat emitted from the product was divided into areas with a uniform amount of heat using CVD, and cooling channels were created toward the center of mass of each area to imitate plant growth.

**Figure 1:**
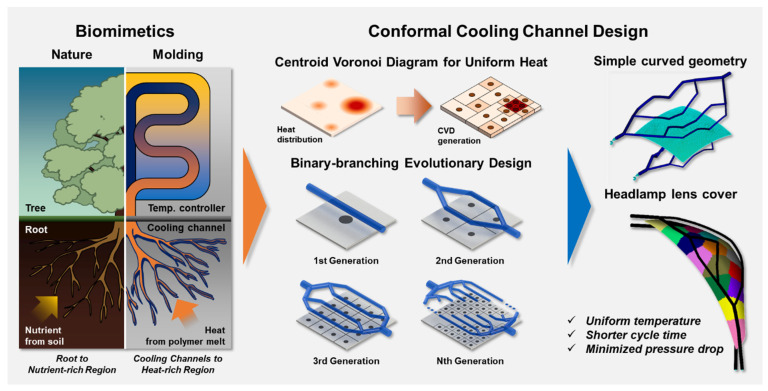
Schematic of biomimetic conformal cooling channel design method.

In addition, as the roots of plants grow over time, the concept of evolution of the cooling channel was introduced by mimicking the generation of small-diameter roots from large-diameter roots. After the cooling channel was fabricated within one generation, the diameter, depth, and flow rate of the cooling channel were optimized as design variables. If the temperature deviation value as the objective function has not reached a certain temperature threshold after optimization, it evolves to the next generation, and all cooling channels created in the previous generation were deleted. In the next generation, the cooling channel was subdivided and installed based on the cooling channel of the previous generation, and the same optimization process was repeated. Using this methodology, the cooling channel continued to evolve until it reached the objective function. The schematic of this approach is Figure 1.

The evolution of the cooling channel was automated through the interlocking with PIAnO (PIDOTECH Inc., Seoul, Republic of Korea), an optimization software. The performance curve of the pump was used to consider the actual state of the cooling equipment, such as a mold temperature controller. The range of the required flow rate and diameter of the cooling channel can be calculated by the performance curve of the pump presenting the flow rate corresponding to the head.

The above process requires one to link three pieces of software: PIAnO; Moldflow (Autodesk Inc., San Francisco, CA, USA) for injection molding analysis; and Matlab (MathWorks Inc., Torrance, CA, USA) for path calculation during cooling channel generation. The design of the cooling channel and heat transfer analysis was performed using Moldflow providing a well-developed application programming interface (API) for linking with external software. Initial verification was performed on an arbitrary curved surface, and then the improved methodology was applied to a lens cover of automobile headlamps. Finally, the reliability of the proposed approach was verified by implementing the design results in Fluent (ANSYS Inc., Canonsburg, PA, USA), a piece of computational fluid dynamics (CFD) software.

Using the above methods, an attempt was made to minimize the flow stagnation phenomenon inside the cooling channel, which was not considered in previous studies. Additionally, we attempted to increase the practicality by considering the pump performance of the cooling system used when cooling the actual mold, rather than the theoretical design theory.

## 2. Method to Design and Evolutionary Cooling Channel

**Figure 2:**
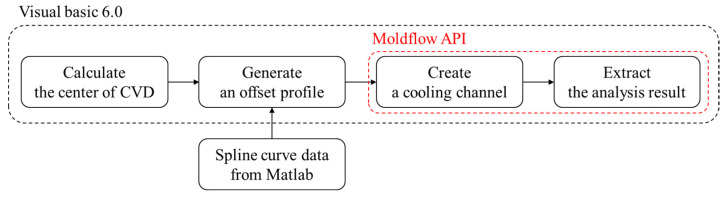
Flowchart for designing the cooling channel.

In this study, the concept of evolution was introduced by mimicking the growth of plant roots. In the first generation, CVD is used to calculate the center of mass on the surface of the product, and only one cooling channel passing through it is created. The method of creating a cooling channel was automated by Moldflow API. After generating an offset profile separated by a certain distance on the product surface, a cooling channel is created by B-spline curve following the created points [16]. The flowchart of this methodology is represented in Figure 2.

The objective function was defined as the standard deviation of temperature to minimize product deformation. The flow rate, diameter, and depth of cooling water were selected as design variables for the first generation. To design a cooling channel, it is necessary to determine the diameter, flow rate, and depth of the cooling channel. However, in the concept of evolution of the cooling channel, all design factors were defined as design variables because the cooling channel was designed to produce optimal efficiency by mimicking plant roots. Since the flow rate was derived from the heat transfer equation in the first generation, it was defined as a design variable only in the first generation to verify the induction process during optimization.

Using the described design variables, the experimental points were extracted using the Central Composite Design (CCD) method. An approximate model using the Kriging method was generated, and optimization was performed using Progressive Quadratic Response Surface Method (PQRSM) and PIAnO [17]. If the objective function value was not satisfied in the first generation, the created cooling channel was defined as the parent cooling channel before branching for the next evolving generation.

The diameter before branching was used as the first-generation optimal value because it is difficult to define a rule for optimization if the limiting range of design variables changes every time optimization. Therefore, the range of design variables calculated in the first generation continued to be used the same after evolution.

**Figure 3:**
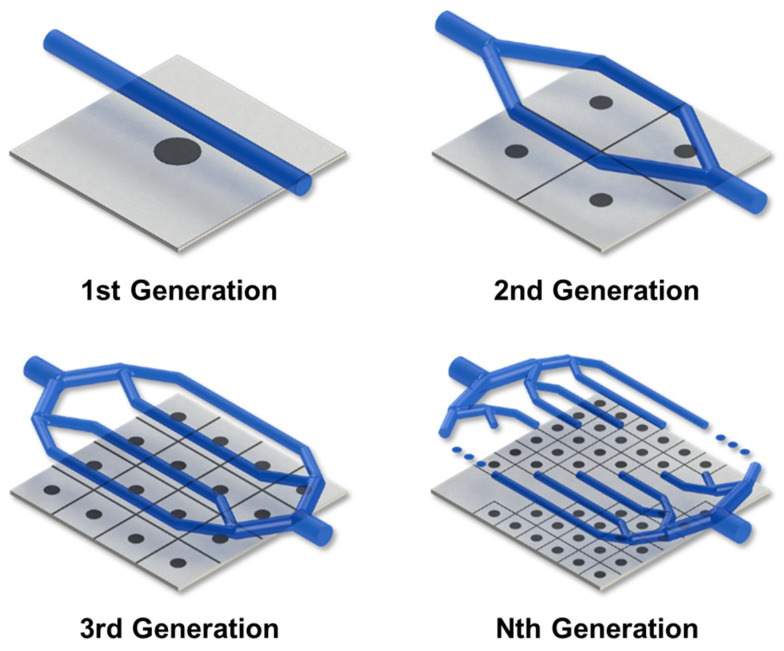
Schematic of the evolution process of the cooling channel.

In the second-generation cooling channel, as shown in Figure 3, the cooling channel is branched by the binary branching rule, and the cooling channel after branching is defined as the child cooling channel. In this case, four regions are created using CVD, and a cooling channel is created around each region.

As in the previous generation, the diameter and depth of the cooling channel are used as design variables. However, only one child cooling channel diameter for each of the upper and lower sides is used as a design variable since the relationship between the parent and child cooling channel is derived from Murray’s law. The depth of the cooling channel was selected in succession on the upper and lower sides regardless of the generation. Finally, after fixing the diameter of the parent cooling channel optimized in the previous generation for the cooling water circulation structure, only one inlet and outlet for the cooling water circulation structure were used.

If the cooling channels are branched up to the *N*th generation in the above manner, the total number of child cooling channel diameters is $2\times 2^{\left(N-1\right)}$, and the product area is divided into $4^{\left(N-1\right)}$ regions, as shown in Figure 3.

### 2.1. Murray’s Law

**Figure 4:**
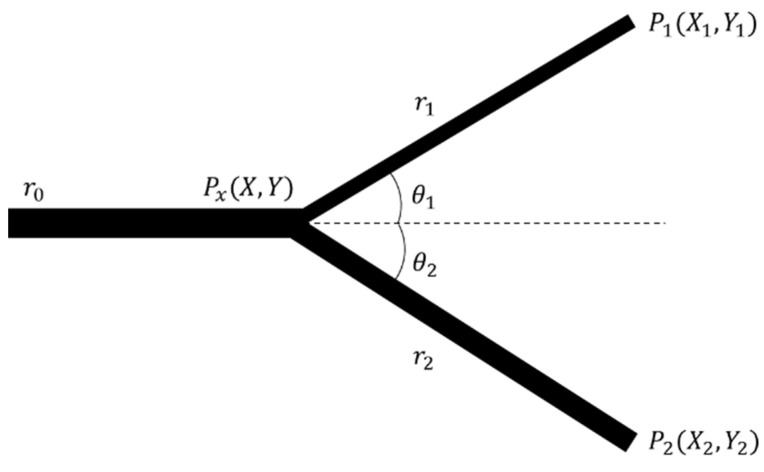
Cooling channel junction determination methodology.

The diameter was determined using Murray’s law for branching one parent cooling channel to two child cooling channels. Murray proposed the relationship of the diameter of the blood vessel branches minimizing the flow resistance of blood flow supplied from the human heart. Therefore, it was expected that the flow resistance could be minimized in the same manner in the cooling channel. Since the branch point cannot be calculated using Murray’s law, the branch point minimizing the flow resistance was evaluated according to Fung’s study, as shown in Figure 4 [18].

(1) $$\theta_{1}=\cos^{-1}\left(\frac{r_{0}^{4}+r_{1}^{4}-r_{2}^{4}}{2r_{1}^{2}r_{2}^{2}}\right),\;\theta_{2}=\cos^{-1}\left(\frac{r_{0}^{4}-r_{1}^{4}+r_{2}^{4}}{2r_{1}^{2}r_{2}^{2}}\right)$$

(2) $$P_{x}=\left[\begin{array}{c} X\\ Y \end{array}\right]={\left[\begin{array}{cc} \tan\theta_{1} & -1\\ \tan\theta_{2} & -1 \end{array}\right]}^{-1}\left[\begin{array}{c} X_{1}\tan\theta_{1}-Y_{1}\\ X_{2}\tan\theta_{2}-Y_{2} \end{array}\right]$$

Here, $P_{x}$ denotes the branching start point to be calculated. $r_{0}$ defines the diameter before branching, and $r_{1}$ and $r_{2}$ define two diameters after branching, respectively. $P_{1}$ and $P_{2}$ are the calorific center points of the region calculated by CVD. $X_{1}$, $Y_{1}$, $X_{2}$ and $Y_{2}$ are coordinates of $P_{1}$ and $P_{2}$. $\theta_{1}$ and $\theta_{2}$ are relative angles to the previous channel before branching, respectively.

After calculating the $\theta_{1}$ and $\theta_{2}$ values using Equation (1), the calorimetric center point value extracted using CVD and calculated $\theta_{1}$ and $\theta_{2}$ values are substituted into Equation (2) to calculate the branch point coordinates.

### 2.2. Centroidal Voronoi Diagram (CVD)

CVD was used as a method for calculating the area emitting the same amount of heat from the part. The Voronoi Diagram (CV) is defined as an area comprising only the points closest the arbitrary seed point, as described by Equation (3).

(3) $$V_{i}=\left\{w\in Ω\;|\;d\left(w,z_{i}\right)<d\left(w,z_{j}\right)\right\}\;\mathrm{for}\;j=0,\;1,\cdots ,n-1,\;\mathrm{and}\;j\neq i$$

Here, w is the set of all points in the Euclidean space. $V_{i}$ represents Voronoi cell, or Voronoi region, associated with the site $z_{i}$, which is the set of all points in Ω, whose distance to $z_{i}$ is not greater than their distance to the other sites $z_{j}$, where j is any index different from i. Ω denotes metric space with distance function d(x, y), which means distance between x and y.

**Figure 5:**
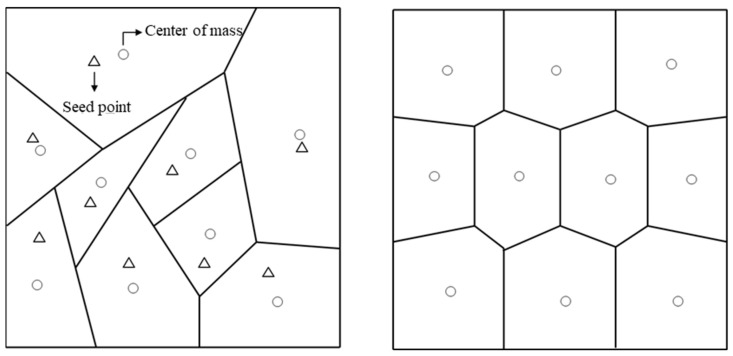
Voronoi Diagram (**Left**) and Centroidal Voronoi Diagram (**Right**).

Unlike VD, the seed point and center of mass of the generated CVD region coincide, as shown in Figure 5. Equation (4) was used for the CVD generation to increase the uniformity of the mesh size using CVD [19]. Since the previous calculation used three-dimensional coordinates, it had the advantage of being immediately applicable to this study.

(4) $$Minimize\left(E\right)=\overset{n-1}{\underset{i=0}{\sum }}\rho \left(x\right)\|x-z_{i}\|{}^{2}dx\;$$

where ρ(x) is a density function of $V_{i}$, and E means energy to construct CVD.

## 3. Definition of Design Variables

### 3.1. Determination of the Required Flow Rate of the Cooling Channel

The flow rate, diameter, and depth of the cooling channel were defined as design variables for optimization. Thus, the limit range of each design variable had to be calculated. First, the flow rate was calculated by Equation (5) presenting the minimum required flow rate of cooling water to remove the emitted heat from the part [20]. The calculated flow rate was defined as the minimum flow rate to prevent heat accumulation and low cooling efficiency.

(5) $$\dot{m_{c}}=\frac{C_{p}m_{p}\left(T_{melt}-T_{eject}\right)}{C_{c}t_{c}\left(T_{inlet}-T_{outlet}\right)}$$

Here, $C_{p}$ and $m_{p}$ indicate the specific heat and mass of the injected polymer, respectively. $T_{melt}$ and $T_{eject}$ represent the melt temperature and ejection temperature of the polymer, respectively. $t_{c}$ represents the cooling time. $C_{c}$ and $\dot{m_{c}}$ mean the specific heat and flow rate of the cooling water. $T_{inlet}$ and $T_{outlet}$ are inlet and outlet temperature of the cooling water, respectively.

The maximum flow rate was determined using the performance curve of the internal pump of the cooling system to be used in the cooling channel. Mass-production factories have standardized cooling water supply equipment. If the flow rate of the equipment is out of the limit, the cooling efficiency will inevitably decrease, regardless of the design of the cooling channel.

**Figure 6:**
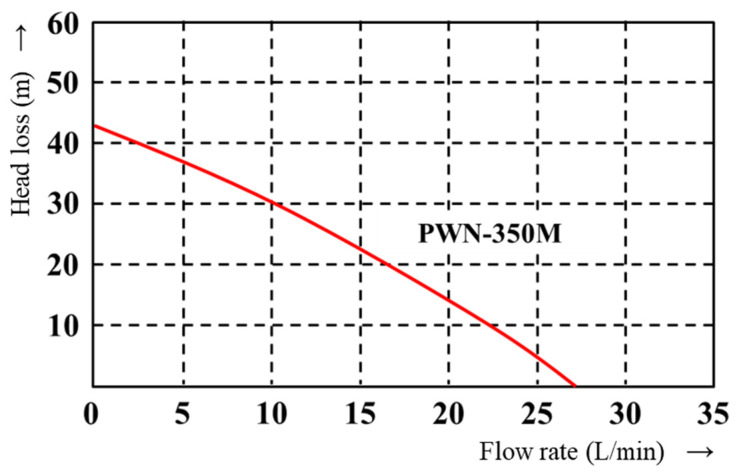
Pump performance curve.

Figure 6 shows the pump performance curve of the pump PWN-350M in Regloplas 150 KL temperature controller (Regloplas AG, St. Gallen, Switzerland) selected for this study. Referring to the graph, the maximum flow rate reaches approximately 27 L/min at 0 m of head (pressure) loss inside of the cooling channel. Therefore, a flow rate of 27 L/min or more acts as a non-discharging flow rate. Thus, this value was defined as the maximum flow rate.

### 3.2. Determination of the Diameter and Depth of the Cooling Channel

The minimum value of the diameter of the cooling channel was determined according to Equation (6) [20].

(6) $$D=\sqrt[{4}]{\frac{16\rho_{c}{\dot{Q}}_{c}^{2}}{7200\pi^{2}\Delta p}\left[f_{c}\left(\frac{L_{c}}{D}\right)+n_{c}K_{c}\right]\;}$$

Here, D denotes the diameter of the cooling channel, $\rho_{c}$ and ${\dot{Q}}_{c}$ denote the density and flow rate of cooling water, respectively. $f_{c}$ and $n_{c}$ denote the length and the number of bends of the cooling channel. $K_{c}$ denotes the resistance coefficient of the bent part, and Δp denotes the pressure drop value inside of the cooling channel. The maximum pressure loss value inside of the cooling channel was calculated as a limiting condition so that the minimum diameter value can be calculated with $n_{c}$ = 0, assuming that only one straight cooling channel is installed.

For the maximum diameter of the cooling channel, Equation (7) was used to calculate the Reynolds number in the tube. Generally, when the Reynolds number exceeds 10,000 in a pipe flow, it is considered a fully developed turbulence. Thus, the diameter maintaining this value was defined as the maximum diameter [21]. μ denotes the viscosity of the cooling water, $V_{c}$ denotes the average velocity of cooling water, and $A_{c}$ denotes the area of the cooling channel.

(7) $$Re=\frac{4\rho_{c}\dot{Q_{c}}}{\pi \mu D}>10,000$$

(8) $$V_{c}=\frac{\dot{Q_{c}}}{A_{c}}$$

(9) $$\;A_{c}=\pi \frac{D^{2}}{4}$$

The depth of cooling channel was one factor that significantly influences the cooling rate. However, the cooling channel cannot be created too close to the mold surface due to the structural stability of the mold. Since the cavity pressure generated during injection molding instantly reaches several tens of MPa, the wall of the cooling channel and mold should have sufficient thickness to withstand the injection pressure, and cracks. Thus, the depth of a typical cooling channel is designed to be at least twice the diameter [20]. Concerning this, the minimum depth of a cooling channel is defined as twice the diameter. In the case of the maximum depth, the cooling efficiency decreased rapidly due to the farther distance from the product to the cooling channel.

## 4. Verification of Evolutionary Cooling Channel

### 4.1. Specimen Shape Verification

**Figure 7:**
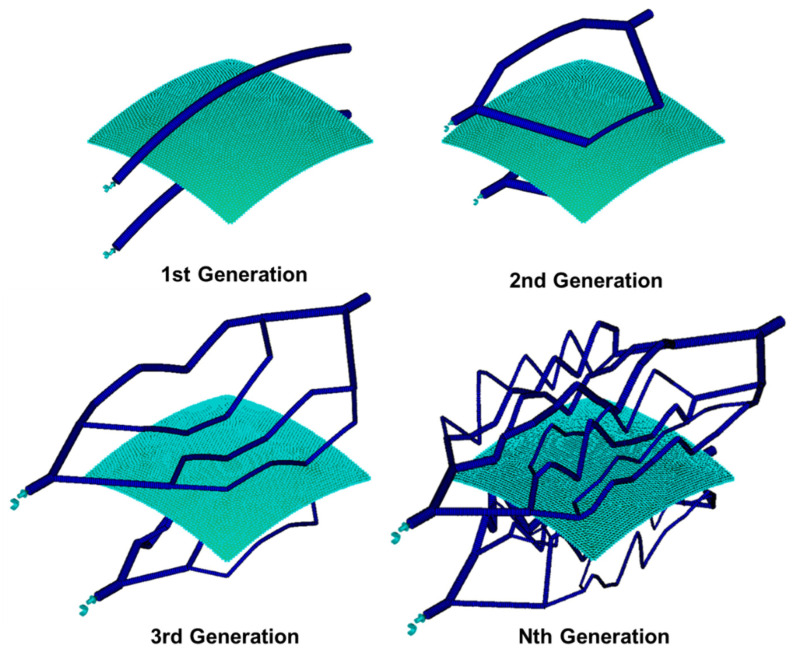
Evolution result of the cooling channel with specimen shape.

**Figure 8:**
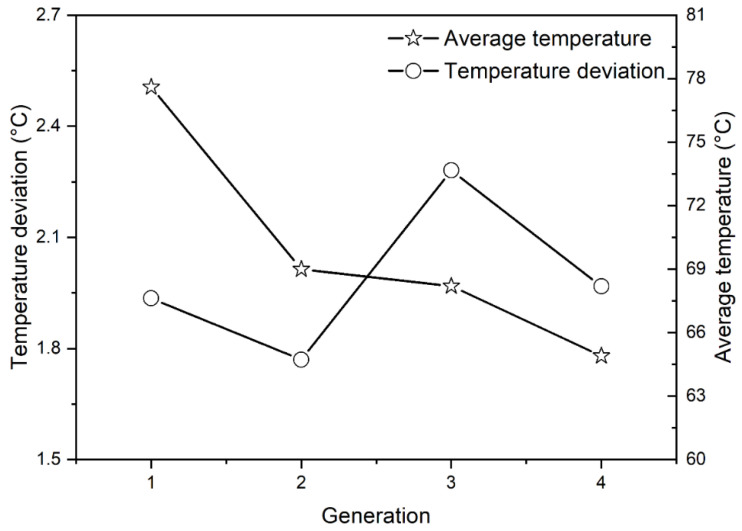
Standard deviation of temperature and average temperature change graph by generation.

Before applying the actual product, the methodology was verified using a rectangular curved surface geometry. Since the temperature deviation value was not significant in this simple shape, it was forced to evolve up to the fourth generation. The temperature deviation and additional effects were checked. Figure 7 shows the generated cooling channels as the cooling channel evolution, and Figure 8 shows the temperature deviation and average temperature.

Figure 8 confirms that the average temperature continued to decrease as the cooling channel evolved. However, the temperature deviation decreased until 2nd generation and then increased at 3rd generation. This resulted from the effect of excessive local cooling, and it meant that the deviation value was more suitable than the average temperature value to determine the cooling channel performance.

### 4.2. Head Lamp Lens cover Shape Verification

**Figure 9:**
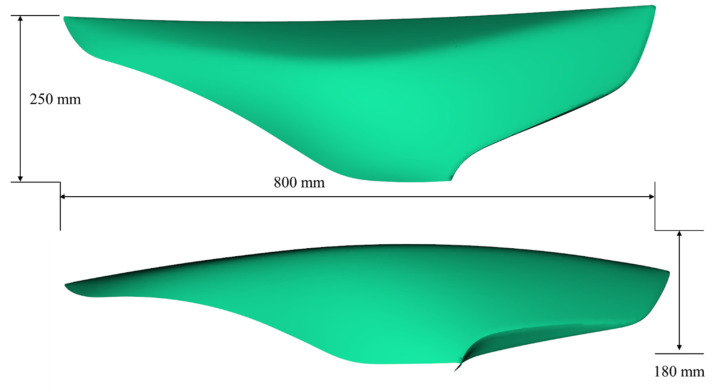
Car headlamp lens cover shape with geometry information.

After verification using the rectangular curved surface geometry, the proposed cooling channel design method was applied to an automobile headlamp lens cover. The product’s shape and geometry are shown in Figure 9. The material was polycarbonate (PC) (Staren ER-1000, Samsung SDI, Yongin, Republic of Korea). Generally, it is difficult to achieve sufficient cooling performance with only a straight-line cooling channel due to the many curved surfaces in the product shape.

Table 1 summarizes the diameter and depth information for the calculation of the minimum flow rate of the evolutionary cooling channel design. The standard deviation of temperature as the objective function was set to 5 °C. This is a limiting value generally used in the mass production process. The cooling channel evolution stopped when this value was reached [20].

**Figure 10:**
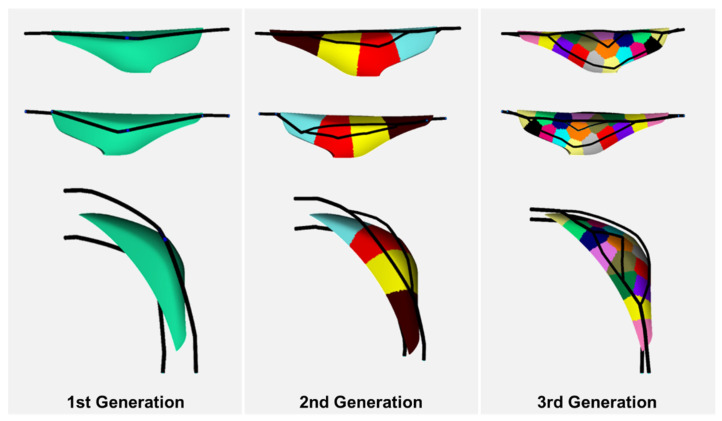
Cooling channel evolution result.

Figure 10 shows the result of the cooling channel evolution confirming that the third generation satisfied the objective function. In the first generation, only one cooling channel was installed. In the second generation, two child cooling channels were branched from one parent cooling channel, so two child cooling channels were installed. The color displayed on the product represents the generated CVD, and since it is the 2nd generation, a total of four CVDs were generated. In the third generation, four child cooling channels were installed, and 16 CVDs were generated. However, only three child cooling channels were installed due to the lack of the center points.

## 5. Analysis Results

### 5.1. Part Temperature Analysis

**Figure 11:**
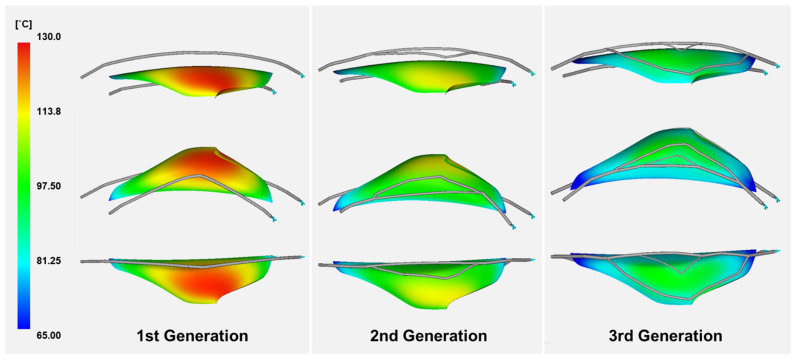
Part temperature results through the evolution process.

The part temperature distribution after the evolution of the cooling channel is shown in Figure 11. In the first generation, a high temperature was generated in the center of the product. This was because only a single cooling channel was installed, so it could not exhibit sufficient cooling efficiency. The cooling efficiency was increased relatively in the second generation, and low temperatures were displayed throughout the product with sufficient cooling efficiency in the third generation.

### 5.2. Temperature Deviation and Pressure Drop Analysis

**Figure 12:**
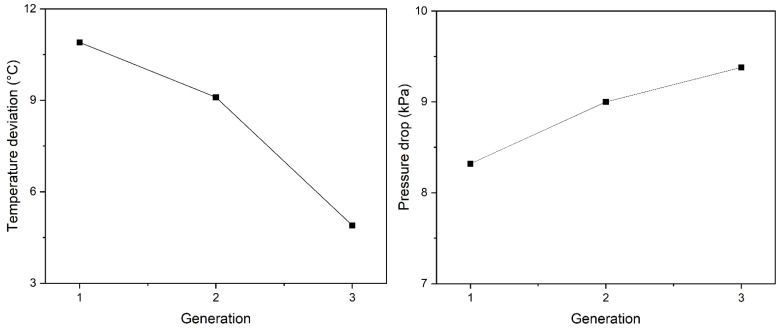
The standard deviation of temperature by generation (**Left**) and pressure drop inside of the cooling channel (**Right**).

The change in the standard deviation of part temperature for each generation is shown in the left of Figure 12. The second generation of cooling channels exhibited 17% of the reduction in the standard deviation of part temperature, comparing to the first generation. The third generation showed 46% of improvement of reduction in the standard deviation of part temperature. In the third generation, a cooling channel created to cover a wider area of the product emitted relatively more heat. Therefore, the deviation of the part temperature was reduced, and it resulted in higher cooling efficiency.

**Figure 13:**
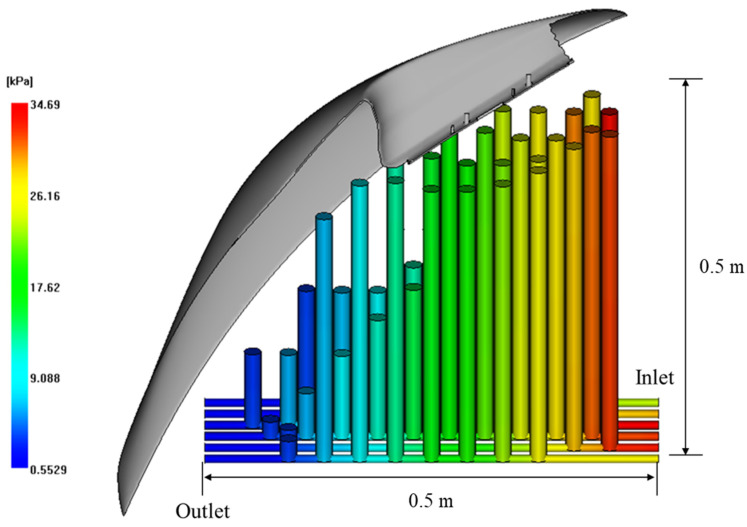
The pressure drop results using the straight-line cooling channels.

To compare the cooling efficiency to the straight-line cooling channel, the pressure drop inside of the cooling channel was checked, as shown in the in the right of Figure 12. Although the cooling channel was subdivided into child cooling channels, the total pressure drop generated in the cooling channel was <10 kPa. Figure 13 represents the pressure drop results of core side using the straight-line cooling channel with the same temperature deviation. The sum of the pressure drops in all cooling channels is over at least 100 kPa. This was because the shape of the product was deep and wide, so the conventional cooling channel design required a lot of baffle pipes, leading to a higher pressure loss.

### 5.3. Temperature Deviation Analysis by Area

**Figure 14:**
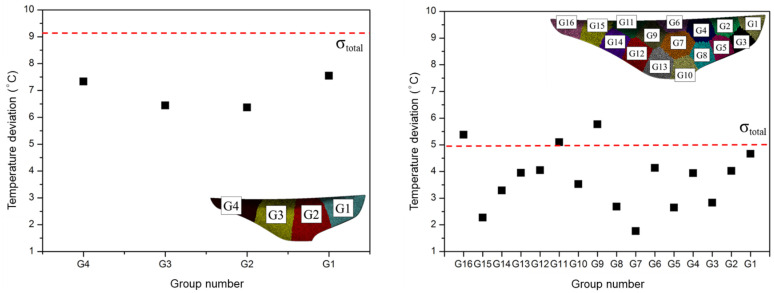
The standard deviation of temperature by area and overall of the second (**Left**) and third (**Right**) generation.

Figure 14 shows the standard deviation of the part temperature of each CVD area, and overall temperature deviation, $\sigma_{total}$. From the results of the second generation on the left in Figure 14, the cooling efficiency is not sufficient, as both $\sigma_{total}$ and the temperature deviation for each area were above the target objective value of 5 °C. However, from the results of the third generation on the right, the temperature deviation of most areas, including $\sigma_{total}$, was 5 °C or less. Therefore, the cooling channel evolution based on the segmented regions effectively improved the cooling efficiency.

### 5.4. Verification of Flow Rate Using CFD

The proposed method of cooling channel evolution was developed using Moldflow. To improve calculation speed, Moldflow used a method of estimating pressure loss by assuming a cooling channel as a one-dimensional cylindrical tube, which might not be accurate for calculating the cooling water flow inside the cooling channel. To verify the reliability of the values obtained through Moldflow analysis, flow analysis inside the cooling channel was performed using 3D CFD analysis, Fluent.

The third-generation cooling channel at the core side was selected as the shape used for verification. After 3D modeling, the 3D cooling channel model was used as the analysis shape in CFD. After generating approximately three million grids, the flow conditions in the cooling channel were set to a mass flow inlet, and the k-epsilon turbulent model, which was the same as the Moldflow boundary conditions, was used.

**Figure 15:**
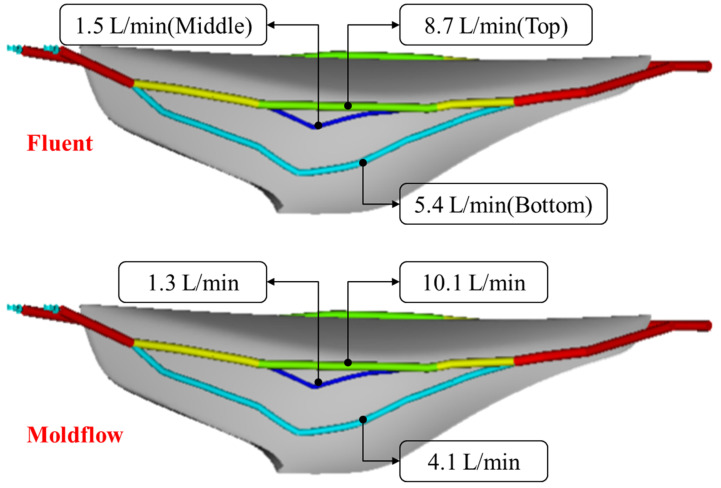
Flow comparison through Moldflow and Fluent analysis in the third generation of cooling channel at the core side.

According to Figure 15, there was a clear difference in the flow rate at the same location, although the tendency was similar. For detailed analysis, the diameter of cooling channels and resulting flow velocity were summarized. By location, the top cooling channel, middle cooling channel, and bottom cooling channel were defined, and the indicated values are shown in Table 2.

In Table 2, the difference in flow rate between the CFD result and Moldflow is shown as a percentage. The CFD analysis result exhibited 20% less, 6% more, and 22% more flow velocity at the top, middle, and bottom cooling channels, respectively. To check the influence of these differences between CFD and Moldflow results, the convective heat transfer coefficient directly affecting the heat transfer amount was checked using Equations (10) and (11). The 22% of difference in flow velocity induced a 12% of difference in the convective heat transfer coefficient [23].

(10) $$Nu=\frac{hD}{k_{c}}=0.0235Re^{0.8}Pr^{0.3}$$

(11) $$h=0.0235\frac{k_{c}}{D}Re^{0.8}Pr^{0.3}$$

Here, Nu and Pr are the Nusselt number and Prandtl number, respectively. $k_{c}$ is the thermal conductivity coefficient of water. h is the convective heat transfer coefficient.

Since the convective heat transfer coefficient is linearly proportional to the convective heat transfer amount by the cooling water, the actual heat transfer amount can be seen to differ by approximately 12%. However, the maximum temperature of the cooling water used in injection molding has not exceeded approximately 90 °C. According to Newton’s law of cooling, even if the maximum cooling water temperature is 90 °C, a 12% difference in heat transfer coefficient has the same heat transfer amount as a change within a range of 10 °C of cooling water temperature. This temperature difference of the cooling water is within the fluctuation range of the melting temperature (approx. 200 °C) of polymer material for injection molding [24]. It means that it does not significantly affect the cooling process. Therefore, Moldflow cooling analysis is reliable in a negligible range because even a flow rate difference of up to 22% occurs in the cooling water, and the effect on the actual heat transfer amount is low.

## 6. Conclusions

This study proposed a design methodology for a conformal cooling channel minimizing the stagnation of cooling water by mimicking the growth of plant roots. The design of the cooling channel was developed considering the performance of the mold temperature controller pump. The binary branching rule was used for the cooling channel so that two child cooling channels were differentiated from one parent cooling channel. The methodology was verified by applying to a rectangular curved surface and an automotive headlamp lens cover.

It was confirmed that the average deviation of the temperature as the objective function was improved by approximately 46%. Additionally, the pressure loss was effectively minimized compared to a straight cooling channel. The flow rate results were verified by 3D CFD analysis. As a result of CFD analysis, it was found that there was a difference in the flow rate value. However, the effect on the cooling process was within a negligible range.

In the future, we plan to focus on two areas that need improvement. The first is to reduce the number of analyses, which rapidly increases as the cooling channel evolves. This is because all variables are defined as design variables without evaluating the impact of the design variables. The next is to fabricate an actual mold using metal 3D printing technology and conduct an experiment. However, the entire injection mold using 3D printing is costly and time-consuming. To solve this problem, an evolutionary cooling channel was applied to the area requiring local cooling to make a mold using 3D printing. The rest of the mold area was manufactured through general machining, and then a single mold was made through the diffusion bonding process.

## Figures and Tables

**Table 1:** Physical quantities are required to calculate the minimum flow rate [22].

Physical Quantities	Values
Specific heat of polymer ($C_{p}$)	1832.5 J/(kg °C)
Specific heat of water ($C_{c}$)	4180 J/(kg °C)
Density of polymer ($\rho_{p}$)	1191.8 kg/m^3^
Melting Point of polymer ($T_{melt}$)	290 °C
Ejection temperature ($T_{eject}$)	100 °C
Volume of the product ($V_{p}$)	3.76 × 10^−4^ m^3^
Cooling time ($t_{c}$)	18.5 s
Temperature difference of cooling channel inlet and outlet ($T_{inlet}-T_{outlet}$)	5 °C
Average product thickness (s)	0.025 m

**Table 2:** Comparison of analysis results of Moldflow and Fluent.

	Top	Middle	Bottom
Fluent			
Diameter (mm)	12.6	6.4	10.4
Flow velocity (mm/s)	1128 (−20.1%)	729 (+6.0%)	1046 (+22.3%)
Moldflow			
Diameter (mm)	12.6	6.4	10.4
Flow velocity (mm/s)	1364	685	813

## Data Availability

The data presented in this study are available on request from the corresponding author.
